# A Comparison of Surgical Subspecialty Match Rates in 2022 in the United States

**DOI:** 10.7759/cureus.37178

**Published:** 2023-04-05

**Authors:** Samantha M Lavertue, Richard Terry

**Affiliations:** 1 Surgery, Lake Erie College of Osteopathic Medicine, Elmira, USA; 2 Family Medicine, Lake Erie College of Osteopathic Medicine, Elmira, USA; 3 Family Medicine, Arnot Ogden Medical Center, Elmira, USA

**Keywords:** licensure examinations, surgical specialties, residency placement, graduate medical education, match rates

## Abstract

This paper examines the 2022 surgical subspecialty results within the Match hosted yearly by the National Resident Matching Program (NRMP) in the United States. It exists to place medical graduates with post-graduate training programs via an algorithm based on ranked lists provided by both residency programs and individual applicants around the world. This paper compares the match rates between allopathic medical graduates (MDs) and osteopathic medical graduates (DOs). Using published NRMP data and reports from Program Director surveys, we explored possible reasons for match rate differences between the two groups, hypothesizing that lower match rates among DOs could be explained by completion of less volunteerism, research, or curricular activities that may have affected their overall first-choice match rates in competitive surgical specialties. While the data showed that MDs consistently out-matched DOs, the cause was deemed multifactorial as the data did not provide concrete evidence to the contrary. We concluded that more data over time should be collected to understand why osteopathic students do not match as well as allopathic students in surgical specialties.

## Introduction

In 2022, one of every four medical school graduates was an osteopathic medical graduate (DO). Last year, DO students comprised 19% of match applicants, with 17.2% being current United States (US) DO seniors. Overall, 84.9% of DOs matched into their preferred specialty, and 15% did not get their preferred specialty [1]. In comparison, US allopathic medical graduate (MD) seniors made up 46.8% of the match, and 89.6% of them matched their preferred specialty [2]. The difference in match rates by preferred specialty is 4.7%. Below we examine the surgical specialties as a portion of that 4.7% and ask the question: why the difference?

Commonly cited areas of difference between allopathic and osteopathic medical students include research experiences and licensure examinations. Osteopathic schools lack National Institutes of Health (NIH) funding that supports much of the ongoing research at allopathic medical schools. In the past year, the American Association of Colleges of Osteopathic Medicine (AACOM) has mobilized Congress to increase funding of research within osteopathic colleges of medicine above the 0.1% NIH funding they currently receive [3]. Many osteopathic medical students participate in research, but only 12% of their research is funded by outside sources such as the NIH [4]. Lack of opportunity to participate in high-level research may be negatively impacting their match rates; however, this question is only theoretical as there exists no specific data on the matter, and a more specific study of how students complete their research would need to be conducted to answer it.

To remain competitive, after the Graduate Medical Education (GME) merger in 2015, many osteopathic students choose to take both the Comprehensive Osteopathic Medical Licensing Examination (COMLEX) and the United States Medical Licensing Examination (USMLE), both of which are minimum competency, pass-fail examinations. In 2021, 5,365 DO students took the USMLE, adding an additional $3.5 million to their educational expenses. AACOM has initiated legislative action to achieve parity for DOs with H.R.8850, The Fair Access in Residency Act. This bill is designed to create parity between MDs and DOs by ensuring that the COMLEX is considered by all Medicare-funded residency programs [5]. It targets the 36% of program directors who responded in the NRMP Program Director survey that they do not consider the COMLEX; moreover, it levels the playing field in the 70% of programs that require the USMLE when the competencies outside of osteopathic manipulative medicine tested by COMLEX are equivalent [6].

## Materials and methods

Using data collected and published by the NRMP on the outcomes of US MD and DO seniors reported for the 2022 Match, this study was conducted in November 2022. Due to the use of publicly available data published by the NRMP, this study was granted a letter of IRB exemption by the Lake Erie College of Osteopathic Medicine (LECOM) IRB.

The original data were collected from student applications in the Main Residency Match and included factors on match outcome, specialty preference, and ranking information. Self-reported student background information such as COMLEX and USMLE scores, prior work experience, publications and presentations, and volunteer experiences was also used; board scores were verified by the student’s medical school with an intracorrelation coefficient of 0.993 (99% CI: 0.992-0.993) for COMLEX-USA Level 1 scores, 0.994 (99% CI: 0.994-0.995) for level 2-CE scores, 0.999 (99% CI: 0.999- 0.999) for USMLE Step 1 scores, and 0.925 (99% CI: 0.918-0.932) for Step 2 scores. Microsoft Excel was used to create graphic results of the extracted data to make a comparison of these groups [1,2].

Using reports published by NRMP on program director comments and outcomes for both MD and DO students, a literature review was conducted. The program director survey conducted by the NRMP was sent to all participating programs in the Main Residency Match, and the overall response rate was 33.1% (n= 1,507). The survey queried programs about interview and ranking activities, virtual interviewing, and programs’ practices regarding holistic application review. Extrapolated data from these reports were used to create graphic results, making a comparison of matched and unmatched DO and MD seniors in the 2022 match season [6].

## Results

**Figure 1:**
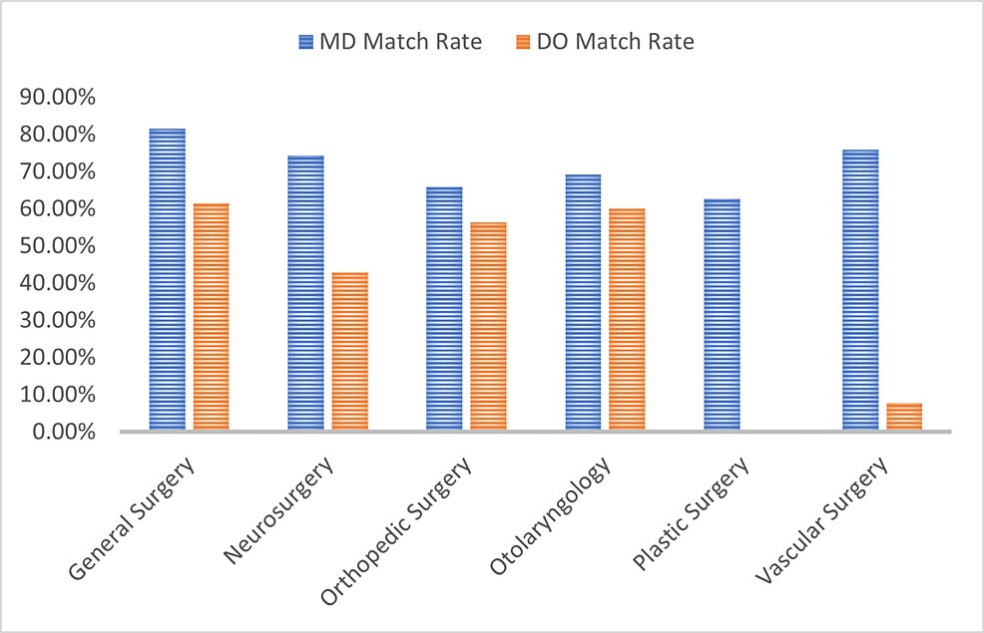
The 2022 surgical match rates by preferred specialty [1,2]

Examined overall by surgical specialty, the 2022 match showed large differences in match rates between MD and DO students. Figure 1 shows these differences.

In general surgery, rates were 81.60% for MDs and 61.50% for DOs. Neurosurgery showed rates of 74.30% and 42.90% for MDs and Dos, respectively. In orthopedics, rates were 65.80% for MDs and 56.30% for DOs. Otolaryngology had match rates of 69.20% for MDs and 60.00% for DOs. Match rates for plastic surgery were 62.7% for MDs and 0% for DOs. Vascular surgery had match rates of 75.80% for MDs and 7.70% for DOs.

Using additional match data about applications, interviews, and match rates for surgical specialties, the percent difference was calculated as shown in Table 1. Here the term “applications” is synonymous with interviews, and match rates are calculated with these values.

**Table 1:** Match rate percent difference by specialty for DO and MD applicants [1,2]

Specialty	MD Applications	MD Matches	MD Match Rate (%)	DO Applications	DO Matches	DO Match Rate (%)	Percent Difference MD vs DO
Neurologic surgery	272	202	73.45	24	9	37.50	35.95%
Orthopedic surgery	1086	705	64.92	205	111	54.15	10.77%
Otolaryngologic surgery	463	316	68.25	41	21	51.22	17.03%
Plastic surgery	281	173	61.57	10	0	0.00	61.57%
Surgery (categorical)	1467	1,059	72.19	397	212	53.40	18.79%
Thoracic surgery	76	41	53.95	4	1	25.00	28.95%
Vascular surgery	100	72	72.00	16	1	6.25	65.75%

When looking at possible reasons for match rate disparities, the 2022 NRMP Program Director Survey provided the following data: of the program directors who answered their NRMP surveys, 29% said that they never interview DOs, and 51% said that they seldom interview DOs. Also, 28% of program directors never rank DOs, and 46% seldom rank them. Results by surgical specialty are compiled into Table 2.

**Table 2:** Program director responses on factors affecting DO applications [5] DO, osteopathic medical graduate; USMLE, United States Medical Licensing Examination; COMLEX, Comprehensive Osteopathic Medical Licensing Examination

Specialty	Never Interview DOs	Seldom Interview DOs	Never Rank DOs	Seldom Rank DOs	Require USMLE Step 2	COMLEX Not Considered
Neurologic surgery	82%	18%	76%	24%	33%	11%
Orthopedic surgery	44%	44%	40%	45%	69%	7%
Otolaryngologic surgery	63%	37%	67%	26%	42%	5%
Plastic surgery	33%	67%	42%	50%	44%	11%
General surgery	32%	50%	24%	57%	58%	11%
Vascular surgery	44%	44%	56%	33%	58%	0%

When looking at possible differences in residency preparation between MD and DO students, we examined the popular categories of research and volunteerism. A comparison was made between both matched and unmatched MDs and DOs, and the results are shown in Table 3.

**Table 3:** Differences in research and volunteerism for matched MD and DO surgical candidates [1,2]

Area of Application by Specialty	Matched MDs	Unmatched MDs	Matched DOs	Unmatched DOs
General surgery				
Mean number of research experiences	4.7	4	3	3.1
Mean number of abstracts, posters, presentations	8.6	5.3	4.6	3.9
Mean number of volunteer experiences	8.9	7.8	7.9	7.5
Neurological surgery				
Mean number of research experiences	6.6	5.9	7.8	2.6
Mean number of abstracts, posters, presentations	25.5	11.7	32.6	7.1
Mean number of volunteer experiences	7.6	7	6.8	7.3
Orthopedic surgery				
Mean number of research experiences	6.6	5.4	4	3.3
Mean number of abstracts, posters, presentations	16.5	12.1	7	4.9
Mean number of volunteer experiences	8.9	7.5	7.2	6.8
Vascular surgery				
Mean number of research experiences	6.3	3.8	5	3.4
Mean number of abstracts, posters, presentations	12.4	11.3	16	4.4
Mean number of volunteer experiences	7.1	6.6	5	7.4

## Discussion

Osteopathic students have a 19% less chance of matching than allopathic students. This percent difference ranged from 10.77% in orthopedics to 65.75% in vascular surgery. The bottom line: DOs are facing match disparities in surgical specialties.

The 2022 NRMP Program Director Survey highlighted some key areas of concern for osteopathic medical students. With specialties such as neurologic surgery admitting that 82% of programs never interview DOs and 18% of programs rarely interview DOs, there is little room for a DO to match. While some programs may occasionally interview DO students, they must then contend with the fact that their own licensing examinations may not be considered. In orthopedic surgery, 69% of programs require USMLE, and 7% of programs will not consider a COMLEX score either alone or in conjunction with the USMLE Step 2 score. These types of percentages all but require DO students to take both examinations to remain competitive. With a common accreditor, the U.S. Department of Education, DOs do double the work and incur twice the cost to meet the same competencies as MDs.

The aforementioned H.R.8850 Fair Access to Residency Act attempts to address the issue of examinations; however, it only ensures parity for DOs in that it would require all Medicare-funded residency programs to consider COMLEX in equal measure to USMLE [7]. This act has been associated with considerable controversy with the American Osteopathic Association (AOA) and the Student Osteopathic Medical Association (SOMA)’s negative position on the bill and the desire to find non-legislative routes to achieve the same result [8]. AACOM and many others support this bill, but the internal strife poses a significant barrier to a bill that will ultimately support parity between MD and DO students [9]. This act, while an important discussion in the setting of a conversation about the DO party, however, is not only unenforceable but also not the real issue at hand when the question of equal opportunities arises. Using vascular surgery as an example, all the surveyed programs consider a COMLEX score even though 58% of programs also require a USMLE Step 2 score, but DO students still had a 65.75% less chance of matching than an MD student. Post-merger, DO students fight for residencies previously set aside just for them, and now they face the odds listed above [10,11].

When other reasons for a difference in match rates are examined, the data provide few answers to DO match disparities. Again, using vascular surgery as an example, the match rate difference was 65.75%, but matched osteopathic students had on average four more research abstract, posters, or presentations than allopathic students. Likewise, with neurological surgery, the match rate difference was 35.95%, but matched DOs had seven more abstracts, posters, or presentations than matched MDs. Without an identifiable pattern, the answer is proven to be not as straightforward as increasing research experiences [1,2].

This study poses several limitations: our data on program directors rely on the overall percentage of program directors who responded to the survey that the NRMP sent. This year, the response rate was only 33.1% (n=1,507 programs). Of the NRMP data on outcomes for both MD and DO seniors, only the students consenting to shared data are used, and therefore, there may be pertinent data missing. There is a limitation in extrapolated data on those who were ranked and matched versus those who were just interviewed as we can only assume that there was some amount of bias without concrete evidence to the contrary.

## Conclusions

The data highlight the fact that osteopathic students are not matching as well by preferred surgical subspecialty as allopathic students. It appears that there may be some elements of discrimination, though the extent is uncertain. The match rate disparity is likely multifactorial, but the data do not show clear areas for improvement, such as volunteerism, research experiences, or publications. Data from program directors, however, clearly indicate that DOs are not interviewed and ranked as often as MDs, which may provide an area for improvement in the system that would allow for high match rates for future osteopathic physicians. One in four practicing physicians is a DO, and one in four residency applicants is a DO. One way to achieve a positive increase in DO match rates would be to interview and rank osteopathic students and allopathic students proportionally. Equal rights to practice should mean equal opportunities to match, but more studies would need to be conducted on the outcome of such a rule.
